# Hydroidfest 2016: celebrating a renaissance in hydrozoan research

**DOI:** 10.1186/s13227-017-0070-1

**Published:** 2017-04-27

**Authors:** Christophe Dupre, Juris A. Grasis, Robert E. Steele, Christine E. Schnitzler, Celina E. Juliano

**Affiliations:** 1 Neurotechnology Center, Department of Biological Sciences and Neuroscience, Columbia University, New York, NY 10027 USA; 2 Department of Biology, San Diego State University, San Diego, CA 92182 USA; 3 Department of Biological Chemistry and Developmental Biology Center, University of California Irvine, Irvine, CA 92697 USA; 4 Whitney Laboratory for Marine Bioscience, University of Florida, St. Augustine, FL 32080 USA; 5 Department of Biology, University of Florida, Gainesville, FL 32611 USA; 6 Department of Molecular and Cellular Biology, University of California, Davis, CA 95616 USA

**Keywords:** Hydrozoans, *Hydra*, *Hydractinia*, Siphonophores, *Aiptasia*, *Cassiopea*

## Abstract

Hydroidfest 2016 took place on September 23–25 at the UC Davis Bodega Marine Laboratory in Bodega Bay, CA. The meeting brought together cnidarian researchers, with an emphasis on those studying hydrozoans, from North America and other parts of the world. The scientific topics discussed were diverse, including sessions focused on development, regeneration, aging, immunology, symbiosis, and neurobiology. Thanks to the application of modern biological technologies, hydrozoans and other cnidarians are now fertile ground for research in numerous disciplines. Moreover, their amenability to comparative approaches is a powerful asset that was repeatedly showcased during the meeting. Here, we give a brief account of the work that was presented and the opportunities that emerged from the ensuing discussions.

## Background

Hydroidfest (www.hydroidfest.org) was organized to promote interactions within the growing community of scientists studying hydrozoan cnidarians, particularly in North America. The meeting took place on September 23–25, 2016, at the UC Davis Bodega Marine Laboratory (BML), in Bodega Bay, California, with 59 researchers representing 7 countries. There were 18 oral presentations in 6 sessions, a keynote address, 16 poster presentations in 2 sessions, and 3 technology workshop speakers. The BML has a long history of hosting scientific meetings aimed at advancing our understanding of marine organisms and helping shape policies related to coastal ecosystems preservation. Accordingly, it provided an ideal environment for scientists to discuss recent advances and plan future collaborations in the numerous fields of research involving hydrozoans.

**Fig. 1:**
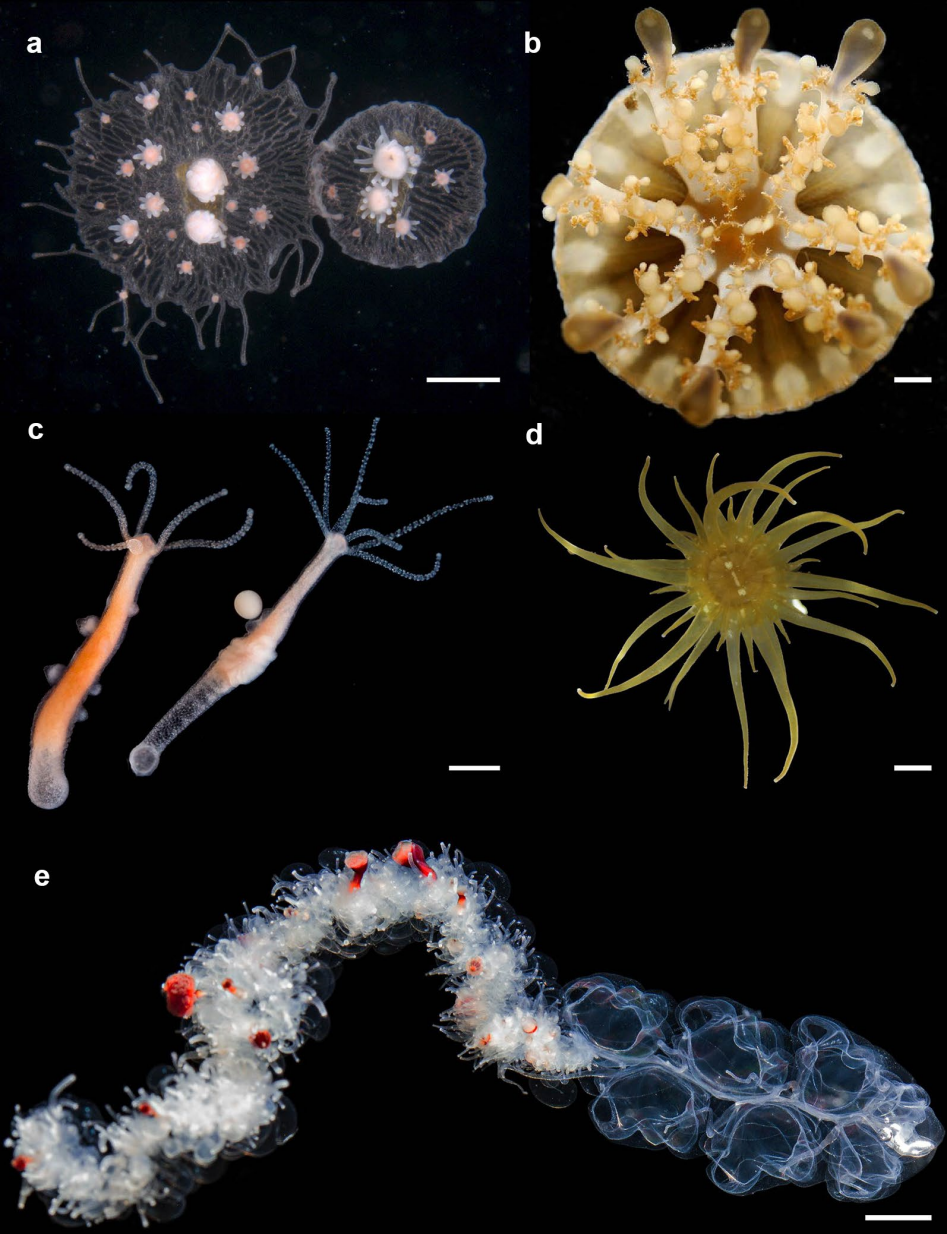
Cnidarians discussed at Hydroidfest. **a** Two colonies of *Hydractinia* that are rejecting each other (courtesy of Matthew Nicotra; *scale bar* 1 mm). **b** *Cassiopea* (courtesy of C. Newkirk; *scale bar* 3 mm), **c** *Hydra* male (*left*) and female (*right*) (courtesy of S. Siebert; *scale bar* 1 mm), **d** *Aiptasia* (courtesy of T. Tivey; *scale bar* 2 mm), **e** Physonect siphonophore *Apolemia sp.* with functionally specialized zooids arranged along the stem of the colony. Anterior part of a colony is shown (Courtesy of S. Siebert; *scale bar* 1 cm)

Hydrozoans are a cnidarian taxon in which the polyp is often the dominant life cycle phase, e.g., in *Hydra* and *Hydractinia*. In addition to hydrozoans, Hydroidfest included additional cnidarian models such as the sea anemone *Aiptasia* and the scyphozoan jellyfish *Cassiopea* (Fig. 1). The remarkable biology displayed by cnidarians has fascinated scientists for hundreds of years [1–4]. For example, *Hydra* research conducted in the middle of the eighteenth century led to the first description of asexual reproduction by budding, the first controlled experiments on animal regeneration, the first successful animal grafts, the first study of phototaxis in an animal without eyes, and the first vital staining of tissues [5]. Although in the past cnidarian studies have been hampered by a lack of molecular tools, recent technical developments are now allowing us to dig deeper into understanding hydrozoan biology and thus offer a bright future for this field. The genomes of *Hydra* [6] and *Hydractinia* (http://research.nhgri.nih.gov/Hydractinia) have been sequenced, and stably transgenic lines of *Hydra* and *Hydractinia* can be created routinely through embryo microinjection [7, 8]. These breakthroughs now enable the use of other modern tools such as CRISPR/Cas9 genome editing, next-generation sequencing, and live fluorescence imaging in hydrozoans, all of which were on display at Hydroidfest 2016.

The scientific research presented at this meeting nicely demonstrated that the same biological properties of cnidarians that both inspired and were instrumental in past research are now poised to play an even more important role when combined with modern technologies. First, translucency is a major asset for fluorescence microscopy, particularly in *Hydra* and *Hydractinia* since they do not have endogenous fluorescent proteins [6]. Second, thanks to asexual reproduction, hydrozoans can be propagated clonally in the laboratory, which alleviates concerns about genetic differences affecting experimental outcomes. Finally, cnidarian plasticity and regeneration are not only important phenomena to study, but are also a great asset to researchers because they allow flexibility in experimental design; researchers are able to study processes in whole animals, or in small samples down to the level of single cells.

A major goal of Hydroidfest 2016 was to strengthen and grow the next generation of hydrozoan scientists. To this end, more than half of the attendees were students and postdocs and these trainees were given ample opportunity to present their research and interact with more senior scientists. The meeting was organized into six sessions of oral presentations, which addressed a wide range of topics in the areas of neurobiology, regeneration, aging, genomes, development, immunology, and symbiosis. There were also two evening poster sessions, and each was preceded by 2-min lightning talks; every poster presenter was thus given the opportunity to present a general overview of her/his material to the entire group. Finally, the meeting ended with a technology workshop where three experts from outside of the cnidarian community covered the topics of genomics, live imaging, and CRISPR-Cas9 genome editing. In this review, we will summarize the research presented in each of the sessions.

## Neurobiology: modeling and imaging

The importance of cnidarian synapses to advance our understanding of neurobiology has been highlighted multiple times [9–12]. Results presented at the conference showed that general questions in hydrozoan neuroscience are ripe to be revisited with modern tools. The hydrozoan nerve net is more complex than initially thought, and it is now clear that a great deal can be learned from researching it.

The laboratory of Rafael Yuste (Columbia University) is using live imaging to answer questions about the organization of the *Hydra* nerve net and the behavior that it creates. Christophe Dupre (Columbia University) is using fluorescent calcium imaging to record the activity of the *Hydra* nervous system, and he has discovered four distinct neural circuits, each of which correlates with a specific behavior. Shuting Han (Columbia University) uses machine learning and computer vision to automatically analyze behavior of *Hydra*. She has developed an algorithm that has allowed her to identify ten behavior types that she has confirmed through manual annotation. Adrienne Fairhall (University of Washington, Seattle) presented her work developing computational tools to address the need for converting large data sets into simpler components, thus making them more manageable for statistical analysis. These tools will allow one to link the activity of the *Hydra* nervous system to specific behaviors.

## Neurobiology: sensing, behavior, and neurogenesis

The nervous systems of hydrozoans have several characteristics of interest to researchers. For example, neurogenesis happens continuously in the adult, and newborn neurons migrate from their site of origin to be incorporated into the nerve net. In addition, despite the lack of specialized sensory organs, hydrozoan neurons detect basic sensory information such as chemicals, mechanical perturbation, and light, which together allow the animal to execute a small and tractable repertoire of behaviors.

James Gahan (National University of Ireland Galway) opened the second neurobiology session by illustrating the power of performing comparative studies with cnidarians. His data show that Notch is important for tentacle patterning but not for neurogenesis in the hydrozoan *Hydractinia*, as opposed to the anthozoan *Nematostella*, which requires Notch for neurogenesis. This difference may be a product of the separate developmental origins of neurons between these two cnidarian species.

David Plachetzki (University of New Hampshire) focused on the evolution of the T1R taste receptors. Although they were thought to be absent from invertebrates, he found that T1Rs are present in cnidarians, and that they co-localize with opsins in *Hydra*. This colocalization allows for both activation and inhibition of cnidocyte discharge and provides new insights into the origins of sensory neurons and their transduction cascades.

Hiroshi Shimizu (King Abdullah University of Science and Technology) discussed the anatomical distribution of neurons in *Hydra* and argued that the higher concentration of neurons in the peduncle could be the precursor of a central nervous system. Accordingly, both wobbling behavior and discharge of adhesive nematocysts could be coordinated by this group of neurons in order to produce locomotion. To study the activity of these neurons, Yukihiko Noro (King Abdullah University of Science and Technology) generated a transgenic *Hydra* line that makes use of the promoter from the Hym-176 neuropeptide gene to express a fluorescent calcium indicator in a subset of neurons located in the peduncle.

## Keynote speaker: Charles David

Charles David (Ludwig-Maximilians-University, Munich) is a scientific leader and inspiring role model in the hydrozoan community. Charlie trained under Max Delbrück and Alfred Gierer and his career spans nearly 50 years of important research accomplishments. Much of our understanding of *Hydra* biology and many of the routinely used methods are a direct outcome of Charlie’s work. His latest breakthrough is the visualization of the entire *Hydra* nervous system using a pan-neuronal antibody developed together with Thomas Holstein (University of Heidelberg). These data led to the discovery that there is no endodermal nerve net in the tentacles, whereas the ectodermal nerve net is present throughout the animal. This tool now makes it possible to study the structure of the *Hydra* nerve net with an unprecedented level of precision and will facilitate efforts to determine how nerve net structure relates to its function.

## Regeneration and aging

Cnidarians are well known for their exceptional regeneration abilities, but not all cnidarians use the same strategy to regenerate. In *Nematostella* and *Hydractinia*, cell proliferation is required for regeneration to proceed, whereas in *Hydra*, newly differentiated structures are built from existing stem cells, without the need for cell division [13–15]. Once again highlighting the power of comparative studies performed within the cnidarian clade, Paulyn Cartwright (University of Kansas) presented her work studying specific gene contributions to these distinct regeneration strategies. She discovered that Runx is involved in *Hydractinia* and *Nematostella* regeneration, but not in *Hydra* regeneration. She therefore hypothesized that Runx is involved in the cell division phase of regeneration.

Stem cells were first discovered in hydrozoans, and these animals remain excellent models for studying stem cell maintenance and differentiation in the context of both regeneration and homeostasis [16]. Stefan Siebert (University of California, Davis) presented his work exploring the function of the PIWI-piRNA pathway in the somatic stem cells of *Hydra*. The PIWI-piRNA pathway is a small RNA regulatory pathway that is best known for germline functions, but has conserved and unexplored function in somatic cells that are being elucidated by work in *Hydra*. Work presented at the meeting suggests that the pathway is required in the somatic stem cells to repress expression of differentiation genes. Interestingly, these somatic stem cells participate in *Hydra* regeneration and future work will aim to uncover how PIWI function impacts regeneration.

Finally, work by Daniel Martínez (Pomona College) and colleagues has demonstrated the absence of senescence (i.e., aging) in *Hydra vulgaris* [13, 17, 18]. Daniel presented research on *Hydra oligactis* which can be induced to senesce by low-temperature induction of sexual reproduction. Approximately 40% of these animals will revert to an “immortal” phenotype after sexual induction; which animals will revert can be accurately predicted before the phenotype is apparent based on the number of testes produced. Thus, Daniel was able to compare gene expression profiles between animals that will revert and animals that will die. Interestingly, he found that transposons are more highly expressed in animals destined to die. This is consistent with observations made in other species where transposon upregulation correlates with aging and may be a causative agent [19]. *Hydra* provides a unique comparative approach to study the mechanisms that underlie transposon upregulation during aging and the effect transposon upregulation has on aging tissues.

## Genes and development

**Fig. 2:**
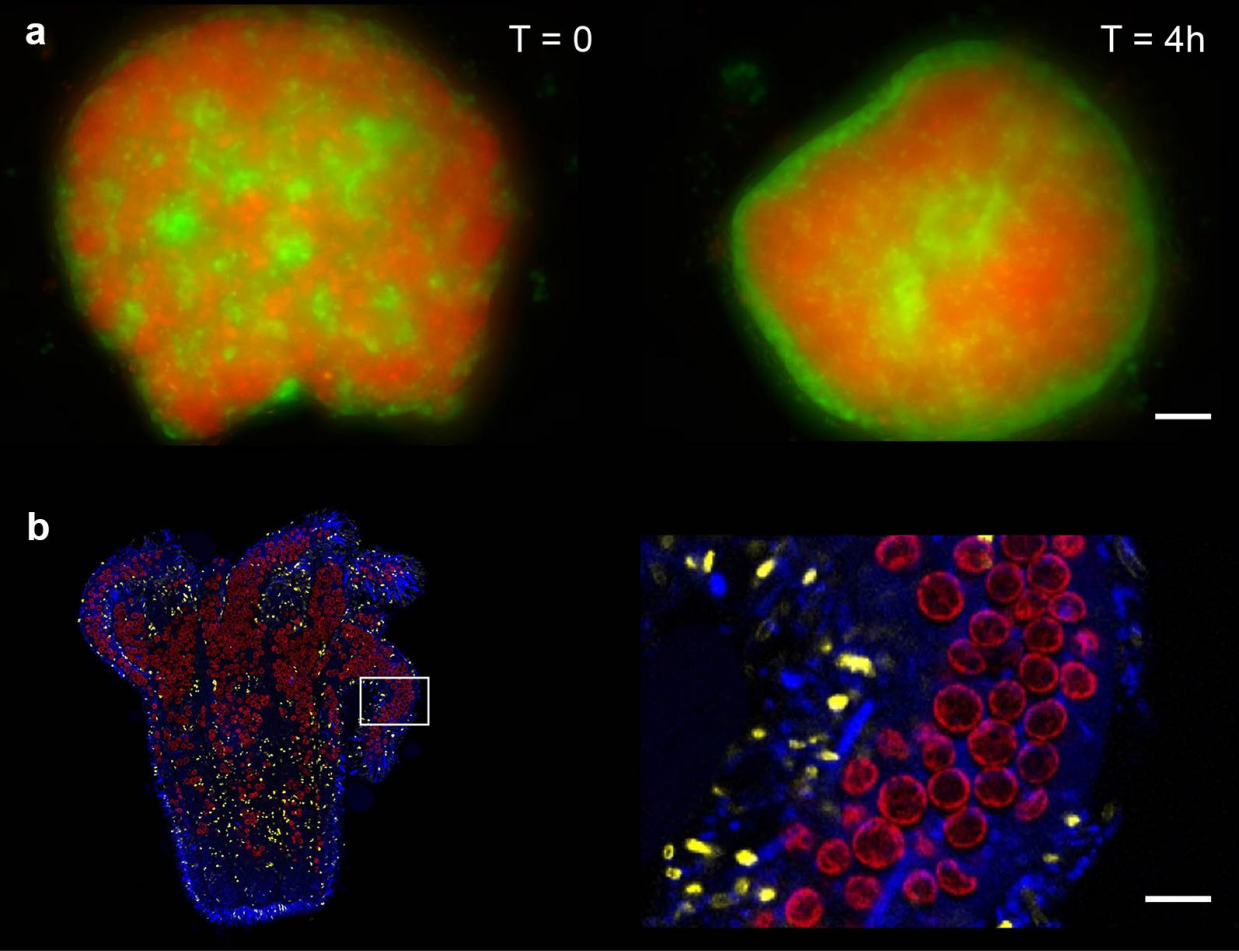
Cnidarians provide unique research opportunities. **a** Following formation of an aggregate from a suspension of *Hydra* cells, endodermal (*red* DsRed2) and ectodermal (*green* GFP) epithelial cells separate into distinct tissue layers. *Left T* = 0 h. *Right T* = 4 h (courtesy of O. Cochet-Escartin; *scale bar* 200 µm). **b** *Aiptasia* with *Symbiodinium* symbionts. *Left* polyp (*blue*, Hoechst staining); symbionts (*red*). *Right* magnified view of the *boxed region* in the *left image* (courtesy of T. Tivey; *scale bar* 15 µm)

The regenerative capabilities of *Hydra* are so powerful that the animal can rebuild itself from a disorganized aggregate of cells [20]. This manipulation offers the opportunity to ask developmental questions that are rarely accessible in adult animals, including the biophysical mechanisms that underlie cellular rearrangements. Using time-lapse microscopy, Olivier Cochet-Escartin (University of California, San Diego) imaged the initial steps of cellular reorganization in aggregates to study how cells sort into distinct epithelial layers (endoderm and ectoderm, shown in red and green, respectively, in Fig. 2a). Olivier aimed to distinguish between two hypotheses: (1) Cellular rearrangement occurs through active cellular mechanisms (e.g., expression of cell adhesion molecules and cytoskeletal reorganization) or (2) Cellular rearrangement occurs through passive mechanisms such as surface tension driving natural separation of the two tissue layers. Olivier’s results favor the involvement of surface tension for multiple reasons, including the fact that the dynamics of cell sorting share similarities with how an emulsion of immiscible liquids separates into distinct layers.

Epithelial cells exhibit planar cell polarity, meaning that cells are arranged in a specific orientation with respect to the underlying extracellular matrix. Using an antibody to detect the localization of the *Hydra* Fat protein (HyFat), Masha Brooun (University of Toronto) demonstrated that *Hydra* epithelial cells exhibit planar cell polarity. HyFat is a cadherin protein that is located at apical junctions of the ectodermal epithelial cells throughout the animal. Masha found that knocking down *HyFat* expression resulted in anatomical abnormalities in epithelial cells. These observations are consistent with a role for HyFat in controlling planar cell polarity in *Hydra*.

This session ended with a presentation of recent progress in sequencing and assembling two *Hydractinia* genomes. Christine Schnitzler (Whitney Laboratory for Marine Bioscience, University of Florida) described a collaborative effort to generate high-quality genome assemblies for *H. echinata* and *H. symbiolongicarpus*. With these data in hand, detailed comparisons with the genome of *Hydra* can now be done. These analyses include searching for regions of synteny among hydrozoan genomes, performing gene content analyses, and comparing non-coding RNA, and repetitive elements. These analyses will provide insight into both novel and conserved features within model hydrozoans.

## Immunology

Hydrozoans utilize a well-conserved innate immune system to detect and respond appropriately to pathogens and symbionts [21]. Microbial recognition leads to antimicrobial peptide secretion and immune response. Additionally, allorecognition is an area of particularly active study in these animals. Hydrozoan research will contribute to our understanding of the evolution of immune responses in animals, and antimicrobial molecules produced by hydrozoans may even be of human therapeutic use.

Steven Sanders (University of Pittsburgh) presented research on allorecognition in *Hydractinia*, which the animal uses to identify a neighboring colony as self or non-self (Fig. 1a). Steve used CRISPR/Cas9 gene editing to study the genes that participate in the underlying process. He generated lines of *Hydractinia* with targeted deletions in the allorecognition gene Alr2, which has been shown to encode a transmembrane protein that undergoes homophilic interactions. These lines made it possible to confirm the role of Alr2 in allorecognition and test which domains of the protein participate in allorecognition.

Kristin Michel (Kansas State University) presented her research testing whether trained immunity exists in *Hydra*. To answer this question, she exposed *Hydra* to low doses of *Pseudomonas aeruginosa*, a gram-negative bacterium, and showed that this exposure, whether with live or heat-killed bacteria, increased the survival time of *Hydra* that were subsequently exposed to a fivefold higher dose of the same bacterium. These data suggest the existence of trained immunity in *Hydra*, but future work is required to understand the molecular underpinnings of these observations.

In addition to possessing an immune system that recognizes microbes, hydrozoans are also capable of producing molecules to regulate these interactions. Jung Shan Hwang (Sunway University, Malaysia) investigates a *Hydra* toxin, HALT-1, which is capable of destroying cells by forming pores in their membranes. Her laboratory is hoping to harness the properties of this toxin to destroy inflammatory macrophages and slow the progress of inflammatory response in human autoimmune diseases such as rheumatoid arthritis.

## Symbiosis

Many cnidarians and dinoflagellates live in symbiosis, which affects the biology of both organisms. An important example of this is the relationship between coral hosts and the dinoflagellates of the genus *Symbiodinium* that live intracellularly within corals [22]. This interaction is vital to coral reef health, but because it is difficult to study corals in laboratory settings, the genes and parameters that play a role in these processes are poorly understood. To gain traction in the laboratory, Trevor Tivey (Oregon State) and Philip Cleves (Stanford University) use the sea anemone *Aiptasia*, which also has a symbiotic relationship with *Symbiodinium* dinoflagellates, to study interactions between a cnidarian and its endosymbiont (Fig. 2b).

Trevor used *Aiptasia* deprived of its symbiont to test the parameters that affect cell division and showed that the absence of the symbiont slowed cell division in the host. Further, he tested parameters such as temperature, light, and nutrition, and found that while feeding increased host size and behavior, there was no difference in cell division between fed and starved hosts.

Phillip determined gene expression profiles following establishment of symbiosis between *Aiptasia* and *Symbiodinium.* He observed changes in both host and symbiont gene expression, and to further examine the roles of these genes, his laboratory generated transgenic lines of *Aiptasia*. This was done using two different strategies: gain-of-function using protein overexpression and loss-of-function using both translation blocking morpholinos and gene knockout with CRISPR-Cas9.

Cnidarians host not only eukaryotic symbionts (such as *Symbiodinium*) but also bacteria and viruses, though the latter are much less well documented than the former. To fill this gap, Juris Grasis (San Diego State University/University of Kiel) presented research that considers the entire holobiont: the functional symbiosis between host, associated prokaryotes and eukaryotes, and viruses. His studies are particularly aimed at finding in which cases the interactions between viruses and *Hydra* are commensal, mutualistic, or parasitic. He found that different species of *Hydra* associate with distinct families of viruses and that these associations have both positive and negative impacts on the metabolism of *Hydra*. Also, he found that *Hydra* uses its innate immune system to regulate its interactions with viruses.

## Technology

The work presented at this meeting relied heavily on genomics, imaging, and gene editing. Therefore, to provide a platform for hydrozoan scientists to discuss new tools in these areas that have been developed in other fields and will prove useful in hydrozoan research, the last session of Hydroidfest consisted of three technology workshops centered on these techniques.

The genome of *Hydra* was sequenced for the first time using a whole-genome shotgun approach [6] and provided an abundant source of data for the entire community [23]. Newer methods promise to help scientists make the most out of the *Hydra* genome by improving the quality of the data that are ultimately made available in repositories. Accordingly, Dan Rokhsar (University of California, Berkeley, Department of Energy Joint Genome Institute, Okinawa Institute of Science and Technology) presented the details of Dovetail Genomics’ in vitro proximity ligation approach for making genome sequencing libraries. Sequencing of such libraries leads to greatly increased genome assembly scaffold sizes. This method has been applied to two hydrozoan genomes, *Hydra* and *Hydractinia*. Dan talked about how larger scaffold sizes make it possible to explore synteny among diverse animal genomes with the goal of determining the organization of chromosomes and arrangement of genes in the genome of the last common ancestor of extant metazoans.

Sara Abrahamsson (Rockefeller University) led the imaging workshop. She is the inventor of a technique that allows imaging multiple planes of a sample simultaneously [24]. This technique relies on an optical device called a “multifocus grating,” which creates an image of each focal plane on a different area of the camera chip. Consequently, the camera continuously collects a 5 × 5 array of images representing 25 different planes of the sample. This drastically increases the imaging speed over the volume of a sample, with a performance that will depend on how transparent the sample is. For this reason, hydrozoans offer a great preparation for this microscope and Sara had the opportunity to image *Hydra* while she was teaching at the Marine Biological Laboratory in Woods Hole, MA, in 2016. She presented her results during the workshop, which triggered excited conversation among an audience eager to know more about modern imaging tools.

Among the multiple techniques available for genome editing, CRISPR/Cas9 has become very popular and is now used in an extraordinarily broad range of species [25]. However, in spite of its widespread use, it is still difficult to streamline experiments using CRISPR/Cas9 because of the numerous parameters that have to be optimized. Gaurav Varshney (Oklahoma Medical Research Foundation) is an expert in the use of CRISPR/Cas9 and developed a high-throughput mutagenesis and phenotyping pipeline to efficiently implement this technology in zebrafish [26]. Numerous steps are involved in this pipeline, and Gaurav’s presentation gave a detailed description of each step, along with potential technical issues and how to overcome them. The first attempts to use CRISPR/Cas9 in hydrozoans are currently underway, and Gaurav’s presentation provided helpful guidance for future implementation, on both small and large scales.

## Poster session and awards

A total of 16 posters (balancing the 18 oral presentations) were displayed outside the conference room for the duration of the meeting. The topics presented were broad and included animal models not covered during the oral presentations, such as siphonophores, and the upside-down jellyfish *Cassiopea*.

*Cassiopea* is a unique model for studying bleaching in cnidarians. Bleaching happens following a stressor such as heat or prolonged absence of light and results in the expulsion of the dinoflagellate symbiont from the host cells. An attractive aspect of the *Cassiopea* model is the possibility to induce bleaching and repopulation of the symbionts. Casandra Newkirk (Whitney Laboratory for Marine Bioscience, University of Florida) is taking advantage of this to measure how gene expression is affected by symbiosis, both in the host and in the symbiont.

Siphonophores are colonial hydrozoans, i.e., they are made of multiple individuals, or zooids, that are connected to each other. Inside an individual colony, all zooids have the same genome. However, each zooid type has a distinct morphology and function. The gene expression profiles associated with each zooid type are thus of interest. Catriona Munro (Brown University) aims to find out how such changes evolved by measuring them in different species.

Krishna Badhiwala (Rice University), Jack Cazet (University of California, Davis), Cawa Tran (Stanford University), and Bryan Teefy (University of California, Davis) were awarded prizes for the best lightning talks sponspored by Eppendorf and Leica. Their research addresses the role of the PIWI-piRNA pathway in wound healing, regeneration and self-renewal of somatic stem cells in *Hydra* (Cazet and Teefy), the use of microfluidics chips to simultaneously record the electrical activity and calcium signals in *Hydra* (Badhiwala) and the role for photosynthesis in the establishment and maintenance of symbiosis in cnidarians (Tran). Rui Wang (University of California, San Diego), Sofia Barreira (National Human Genome Research Institute, National Institues of Health), and Aidan Huene (University of Pittsburgh) each received prizes from Dovetail genomics for their posters on the biophysics of reaggregation in *Hydra* (Wang), repetitive elements in the genome of *Hydractinia* (Barreira), and protein-protein interactions in allorecognition proteins (Huene). James Gahan and Steve Sanders (whose research was described earlier) both received prizes from New England BioLabs for the best oral presentation given by a graduate student and postdoc, respectively.

## Looking ahead: Hydroidfest 2018

It is clear that promising opportunities lie ahead in hydrozoan research and this meeting enabled scientists to exchange ideas, plan future work, and discuss how to address the next challenges. Collaboration will play a crucial role in the future success of hydrozoan research and we are certain that meetings such as Hydroidfest will play a critical role in the formation of such collaborations. Hydroidfest 2018 will take place at the Whitney Laboratory for Marine Bioscience at the University of Florida in St. Augustine, FL. An East Coast equivalent of the BML, this site provides another great venue and a post-meeting survey showed that close to 90% of Hydroidfest 2016 attendees plan to attend in 2018.
